# Comparative effects of low-load blood flow restriction training and high-load resistance training on physical performance in college 800-m runners: a randomized control trial

**DOI:** 10.3389/fphys.2025.1678604

**Published:** 2025-09-30

**Authors:** Jianhua Yu, Jingyan Yu, Lei Zhao, Yi Yang

**Affiliations:** ^1^ Physical Education Department, Nanjing University of Aeronautics and Astronautics, Nanjing, China; ^2^ Physical Education and Sports School, Soochow University, Suzhou, China; ^3^ School of Sport Training, Tianjin University of Sport, Tianjin, China; ^4^ College of Physical Education, Qingdao Hengxing University of Science and Technology, Qingdao, China

**Keywords:** blood flow restriction, 800-m runners, running performance, muscular strengthand power, training load

## Abstract

**Background:**

High-load resistance training (HLRT) is commonly used to enhance performance in 800-m runners but may not always be suitable. Low-load blood flow restriction (BFR) training offers similar benefits to HLRT while reducing these issues. This study aimed to compare the effects of traditional HL-RT and low-load BFR training on muscular strength, power, endurance, and running performance of collegiate 800-m runners over an 8-week training program.

**Methods:**

A total of 22 participants were randomly divided into HL-RT group (n = 11) and BFR group (n = 11). Physical performance was assessed at three time points: baseline, mid-intervention, and post-intervention. The tests included the 20-m sprint test (T20m), countermovement jump test (CMJ), smith machine full-squat test (to assess V1 load), plantar flexion rate of force development test (PF-RFD), 200-m test (T200m), and 800-m test (T800m).

**Results:**

Significant time effects were observed for T20m, CMJ, V1 load, PF-RFD, T200m, and T800m (all P < 0.05), and significant interaction effects between time and group was found for V1load (P < 0.05). Post-training comparisons between the HL-RT and BFR groups revealed a significant improvement in V1 load in the HL-RT group (P < 0.05), while no significant differences were found between the groups for the other performance measures. The present results indicate that both HL-RT and BFR training demonstrate positive effects on the muscular strength, power, endurance, and running performance, while HL-RT demonstrate greater gains in muscular power.

**Conclusion:**

Low-load BFR training offers an effective alternative to traditional HL-RT for enhancing competitive performance and key physical attributes in collegiate 800-m runners.

## 1 Introduction

The 800-m race is one of the most physically demanding events in track and field, requiring a delicate balance of aerobic endurance and anaerobic capacity (Jariono et al., 2024). To enhance athletic performance, resistance training (RT) has become a widely adopted and effective method for improving the conditioning of middle- and long-distance runners. Evidence suggests that RT induces significant neuromuscular adaptations, including enhanced intra- and intermuscular coordination, increased muscle-tendon stiffness, and greater motor unit recruitment and firing rates (Beattie et al., 2014). These adaptations result in improved muscular strength and power, reduced injury risk, and enhanced anaerobic capacity and running economy (Lesinski et al., 2016). RT has been recognized as an important adjunct in traditional running training for runners (Llanos-Lagos et al., 2024). Among various types of RT, high-load RT (HL-RT)—which involves using intensities of 70%–80% of an individual’s one-repetition maximum (1RM)—has been shown to significantly enhance neuromuscular adaptability, maximizing improvements in muscular strength and explosive power (Lesinski et al., 2016; Riscart-López et al., 2024).

However, HL-RT also comes with certain side effects. The high mechanical stress associated with this training, combined with the delayed onset muscular soreness it often induces, can lead to accumulated fatigue, reduced athletic performance, and even overtraining (Scott et al., 2017). As a result, balancing the benefits of HL-RT with its potential side effects has become a key concern for middle- and long-distance runners and their coaches. This has driven researchers and practitioners to explore alternative RT protocols that can offer similar benefits while minimizing side effects.

In recent years, blood flow restriction (BFR) training has gained widespread attention due to its efficiency and effectiveness (Scott et al., 2023). BFR involves performing low-load RT (usually 20%–40% of an individual’s 1RM) while using pneumatic cuffs to partially restrict arterial inflow and completely block venous outflow (Pearson and Hussain, 2015). This creates a localized hypoxic environment within the muscles, leading to the accumulation of metabolic byproducts such as lactate, which induces metabolic stress and cell swelling (Pearson and Hussain, 2015). This physiological condition activates anabolic signaling pathways and enhances the recruitment of high-threshold, fast-twitch muscle fibers, which are typically recruited during high-intensity, high-load exercises (Nielsen et al., 2012). BFR training provides similar benefits to high-load training with lower intensity. However, despite its significant benefits, the potential risks of BFR training should not be ignored. Research indicates that extreme BFR training may cause muscle damage in healthy but unaccustomed individuals, and in some cases, even lead to rhabdomyolysis (Wernbom et al., 2021). Therefore, BFR training should be performed under appropriate conditions with careful supervision. Nevertheless, combining BFR with low-load training provides an effective alternative for athletes who need to reduce training intensity or avoid the strain of high-load exercises.

An increasing body of research has highlighted low-load BFR training as an effective method for enhancing muscular strength and power across various populations (Wernbom et al., 2021; Lixandrão et al., 2018; Vechin et al., 2015; Geng et al., 2024), including individuals with sarcopenia (Zhang et al., 2024), knee osteoarthritis (Ferraz et al., 2018; Wang et al., 2022), and trained athletes (Manimmanakorn et al., 2013). A meta-analysis by Lixandrão et al. even suggests that low-load BFR training is equally effective in increasing muscle mass compared to HL-RT (Lixandrão et al., 2018). Additionally, a meta-analysis by Geng et al. indicated that trained individuals tend to experience greater gains in muscular strength and hypertrophy with BFR, while untrained individuals achieve greater strength gains and similar hypertrophy with HL-RT (Geng et al., 2024). Moreover, BFR traing offers unique advantages over HL-RT, such as enhancing endothelial function (Shimizu et al., 2016), which is closely related to the athletic performance of middle- and long-distance runners.

Although the overall findings on BFR are generally positive, research focusing on its application in trained middle- and long-distance runners, particularly those specializing in the 800-m event, remains limited. Therefore, the aim of this study was to compare the effects of traditional HL-RT and low-load BFR over an 8-week training program on the muscular strength, power, endurance, and running performance of collegiate 800-m runners. The hypotheses guiding this study are as follows: (i) both training groups will show significant improvements relative to baseline performance; (ii) the HL-RT group will demonstrate greater gains in muscular strength and power; and (iii) the BFR group will exhibit more pronounced improvements in 200-m and 800-m race times.

## 2 Methods

### 2.1 Participants

A total of 22 highly trained (Tier 3) male college long-distance runners (Mean ± SD: age 20.4 ± 1.5 years) were recruited through poster advertisements and direct contact with running coaches (McKay et al., 2022). The calculation indicated that a minimum of 17 participants was required. The inclusion criteria were as follows: participants were aged between 18 and 25 years, had at least 2 years of competitive middle-distance running experience, were injury-free for at least 6 months prior to the study, and were actively competing during the collegiate season. All participants were fully informed of the potential risks associated with the study and provided written informed consent. The study was approved by the Ethics Committee of the Sports Science Experiment (approval number: TJUS2025-045).

### 2.2 Study design

A randomized controlled trial (RCT) was conducted for this study. Participants were randomly assigned to one of two groups: a traditional heavy load resistance training group (HL-RT group, n = 11) or a low-load BFR training group (BFR group, n = 11). Randomization was conducted using a computer-generated random number sequence. Both groups performed resistance training twice per week for 8 weeks, totaling 16 sessions each separated by a minimum of 48 h. The HL-RT group engaged in full squats and leg presses with heavy loads, while the BFR group also performed full squats and leg presses, but with a low-load BFR protocol. All training sessions were conducted in a 1:1 setting and were supervised by a trained and qualified member of the research team. The supervising researchers hold exercise prescriptions or physical training certifications issued by the Chinese Society of Sports Science. Participants underwent a series of physical performance tests at baseline (T1), mid-intervention (T2, 4 weeks), and post-intervention (T3, 8 weeks).

### 2.3 Training programs

#### 2.3.1 Heavy load resistance training

The HL-RT training protocol consisted of squats and leg presses, performed using a Smith machine (Multipower Fitness Line; Murcia, Spain) and a leg press machine (Technogym; Italy). Each exercise was completed for 4 sets of 6-8 repetitions at an intensity of 80%–85% of the participant’s current 1-repetition maximum (1RM). Rest intervals of 3–4 min were allowed between sets. During the leg presses and squat exercises, the knee angle was required to reach 90 degrees, with the concentric phase lasting 2 s and the eccentric phase lasting 3 s. To ensure continued progress, the 1RM was reassessed every 2 weeks, and the load was increased by approximately 2.5–5 kg if the participant was able to complete 8 repetitions in all sets. Each 1RM assessment was conducted 48 h before the upcoming training session to allow for adequate recovery. Each session began with a 5-min warm-up that includes light cardiovascular activity and dynamic stretches for the lower body, and ended with 5 min of static stretching targeting the legs and lower back. In addition to the HL-RT, all participants maintained their regular running training (approximately 10–12 h per week) throughout the study period.

#### 2.3.2 Low-load blood flow restriction training

The BFR training protocol performed the same warm-up, stretching routine, and exercises (full squats and leg presses) as the HL-RT protocol, but with different rest intervals and training loads. Specifically, the BFR training protocol consisted of 4 sets: one initial set of 30 repetitions, followed by three sets of 15 repetitions, with 30–60 s of rest between sets (Vechin et al., 2015). The training load was set at 30% of participant’s 1RM, which was also reassessed every 2 weeks. Pneumatic cuffs (10 cm in width) were applied to the most proximal part of both thighs and inflated to 80% of the participant’s arterial occlusion pressure, measured while seated using a handheld Doppler ultrasound (Lixandrão et al., 2015). These cuffs remained inflated throughout all sets and rest periods for each exercise, and were carefully monitored to ensure proper inflation pressure during each training session. In addition to the BFR training, all participants maintained their regular running training (approximately 10–12 h per week) throughout the study period.

### 2.4 Testing procedure and measures

All tests were conducted by the same investigator to ensure consistent measurement quality. The IRM was assessed every 2 weeks. Outcome testing took place at T1, T2, and T3 in the following sequence: 20-m sprint test (T20m), countermovement jumps test (CMJ), smith machine full squat test, plantar flexion rate of force development test (PF-RFD), 200-m test (T200m), and 800-m test (T800m). Participants were instructed to avoid strenuous exercise for 48 h prior to each testing session.

#### 2.4.1 1-Repetition maximum test

The 1RM test was performed using the barbell parallel back squat. Participants warmed up with 5 min of light jogging, followed by 8–10 squats at 50% of their estimated 1RM to activate the muscles, then rested for 2–3 min. During the testing phase, participants performed three attempts with increasing progressively loads. First, they performed 3 squats at 70%–80% of the estimated 1RM, rested for 2–3 min, then performed 1 to 2 squats at 90% of 1RM, ensuring maximum effort and proper form, followed by another 2–3 min of rest. Finally, participants performed the maximal 1RM attempt at 100% or slightly above estimated 1RM. A successful 1RM was recorded if they completed a full squat with proper form. If unsuccessful, participants were allowed to rest, reduce the weight, and attempt again (Schoenfeld et al., 2019).

#### 2.4.2 20-M sprint test

The T20m was conducted on an indoor track using two pairs of photocells (Polifemo Radio Light; Microgate, Bolzano, Italy). The starting position was standardized, with the starting line placed 1 m behind the first-time gate. The photocell gates were positioned at the start and at the 20-m mark, 0.4 m above the ground. Participants began from the starting line and sprinted as fast as possible to the finish line. Each participant completed the test twice, with a 3-min rest interval between attempts. The best sprint time was recorded to evaluate their sprinting ability (Gisladottir et al., 2024).

#### 2.4.3 Countermovement jump test

The CMJ test was performed using an infrared platform (Optojump; Microgate), where participants started from a standing position, keeping their hands on their waist, and after a preparatory countermovement, performed a maximal vertical jump. Each participant completed CMJ test three times, with a 1-min rest between each test. The flight times were measured using a digital timer connected to the platform, and were used to calculate jump height using the formula 1/8×g × t^2^ (where g is the acceleration due to gravity, and t is the time). The best jump height from each test was selected for analysis (Barker et al., 2018).

#### 2.4.4 Smith machine squat test

The smith machine squat test was employed to assess muscular strength and power by evaluating the mean propulsive velocity (MPV) during a full-squat exercise and determining the V1load. The test was conducted using a linear-velocity transducer attached to the Smith machine (T-Force System; Ergotech, Murcia, Spain). During the test, participants were instructed to perform full-squats while moving the barbell as quickly as possible. A full squat required lowering the hips until the thighs were parallel to or slightly below the ground, with the knees not extending past the toes. The test started with a load of 20 kg, which was progressively increased in 5–10 kg increments. There was a 3-min rest after each load increment. MPV was continuously monitored until it dropped below 1 m/s. The load corresponding to an MPV of exactly 1 m/s was recorded as the V1load. If the exact 1 m/s was not identified, we would estimate it using the load-velocity profile. The test concluded once the V1load was identified, and this value was used as the primary indicator of the participant’s dynamic strength and power (Loturco et al., 2023).

#### 2.4.5 Plantar flexion rate of force development test

The PF-RFD test was conducted using a Smith machine equipped with a force plate (Isonet; JLML, Madrid, Spain), with data sampled at 1,000 Hz. In this test, participants performed two sets of five maximal explosive concentric plantar flexion repetitions against a load equivalent to 80% of their body mass. The maximal PF-RFD was calculated by determining the steepest slope of the force-time curve within a 50 ms window, reflecting the participant’s ability to rapidly generate force. The mean value from all 10 repetitions was computed and used for subsequent analysis (Aagaard et al., 1985).

#### 2.4.6 200-m test and 800-m test

The T200m and T800m tests were conducted on an all-weather track using the Brower TCi Timing Systems (HaB International Ltd.). The timing was recorded between two timing gates, with a starting cue provided by the tester. For the T200m test, participants were required to wear spikes and complete two 200-m sprints from a standing position, following a warm-up. A 5-min rest period was taken between the two sprints. The shorter of the two times was used for analysis. The T200m test was conducted on the next day of the T200m test. Participants proceeded to perform the 800-m test from a standing start. To minimize the influence of external factors such as wind speed, we implemented a strict testing protocol, ensuring that all 22 participants were tested within a 3-h timeframe.

### 2.5 Statistical analyses

The sample size was determined using G*Power software, with an ɑ error probability of 0.05, a power of 0.80, and an effect size of 0.74 for muscular strength, which was derived from a systematic review comparing the effects of low-load BFR training and HLRT (Lixandrão et al., 2018). The experimental data were analyzed using IBM SPSS statistical software (version 26.0, Chicago, IL, United States). Descriptive statistics were presented as mean ± standard deviations (Mean ± SDs). Normality of all variables was verified using the Shapiro-Wilk test. Between-group differences at baseline were examined using independent t-tests. A one-way repeated measures analysis of variance (ANOVA) was used to examine the effects of time on performance outcomes. A two-way repeated measures analysis of variance ANOVA was conducted to assess the main and interaction effects for each dependent variable, with factors for group (HL-RT vs. BFR) and time (T1, T2, T3). Post-hoc tests with Bonferroni correction were performed to identify specific differences when a significant interaction effect was observed. Cohen’s d values were calculated and used to determine the effect size, with interpretations categorized as: trivial (< 0.2), small (0.2-0.6), moderate (0.6-1.2), large (1.2-2.0), or very large (>2.0) (Hopkins et al., 2009). Statistical significance was set at the level of <0.05.

## 3 Results

All 22 participants successfully completed the 8-week training program and attended all testing sessions. Adherence to the supervised training sessions was consistently above 95% for both groups, with the HL-RT group at 95.45% ± 5.65% and the BFR group at 96.02% ± 6.42%. Notably, no training-related injuries or adverse events were reported in either group, indicating the program’s safety and effectiveness.

The Mean ± SDs and changes in performance assessment are displayed in Tables 1, 2, 3. The Shapiro-Wilk tests indicated that all data followed a normal distribution. The results of one-way repeated measures ANOVA showed significant main effects of time for all assessed variables: 1RM (F (1, 20) = 11.8, P < 0.05), T20m (F (1, 20) = 11.4, P < 0.05), CMJ (F (1, 20) = 13.1, P < 0.05), V1load (F (1, 20) = 9.8, P < 0.05), PF-RFD (F (1, 20) = 8.5, P < 0.05), T200m (F (1, 20) = 21.8, P < 0.05),and T800m (F (1, 20) = 15.2, P < 0.05). Additionally, significant interaction effects between time and group were observed for V1load (F (Jariono et al., 2024; Lixandrão et al., 2015) = 4.9, P < 0.05) (Table 1).

**TABLE 1:** Measures and comparisons of variables for the HL-RT group and the BFR group at baseline (T1), mid-training (T2), and post-training (T3).

Variables	HL-RT (n = 11)			BFR (n = 11)			Time		Time*Group	
	Pre	Mid	Post	Pre	Mid	Post	F (*P*-values)	Effect size	F (*P*-values)	Effect size
1 RM (kg)	120.5 ± 11.9	133.1 ± 11.5	145.2 ± 12.3	118.6 ± 13.2	127.9 ± 12.8	134.6 ± 13.5	11.8 (0.01)	0. 74	3.0 (0.53)	0.38
T20m (s)	3.15 ± 0.14	3.08 ± 0.12	3.04 ± 0.11	3.18 ± 0.16	3.11 ± 0.15	3.08 ± 0.14	11.4 **(0.01)**	0.72	0.9 (0.41)	0.20
CMJ (cm)	45.2 ± 4.1	47.1 ± 3.9	48.3 ± 4.2	44.8 ± 3.8	46.5 ± 3.7	47.7 ± 4.0	13.1 **(0.01)**	0.77	0.2 (0.82)	0.10
V1load (kg)	75.4 ± 8.2	84.1 ± 7.9	92.4 ± 8.5	74.1 ± 9.1	80.5 ± 8.8	85.1 ± 9.3	9.8 **(0.01)**	0.67	4.9 **(0.04)**	0.47
PF-RFD (N·s^-1^)	4850 ± 510	5120 ± 490	5380 ± 520	4790 ± 540	5050 ± 530	5290 ± 550	8.5 **(0.02)**	0.62	0.1 (0.91)	0.07
T200m (s)	24.4 ± 0.8	23.9 ± 0.7	23.2 ± 0.6	24.3 ± 0.7	24.1 ± 0.6	23.3 ± 0.5	21.8 **(0.00)**	0.99	0.5 (0.49)	0.15
T800m (s)	112.5 ± 3.1	113.2 ± 2.9	110.8 ± 2.5	113.1 ± 2.8	113.8 ± 2.6	111.2 ± 2.4	15.2 **(0.01)**	0.83	0.3 (0.59)	0.12

The bold values denote p < 0.05.

**TABLE 2:** Changes of variables from baseline (T1) to post-training (T3) for HL-RT group and BFR group.

Variables	HL-RT (n = 11)		BFR (n = 11)	
	% change	Within-group *P*-values	% change	Within-group *P*-values
1 RM	20.9 ± 8.4	**0.00**	14.3 ± 13.4	**0.01**
T20m	−3.5 ± 0.8	**0.01**	−3.2 ± 1.7	**0.02**
CMJ	6.8 ± 2.9	**0.01**	6.5 ± 3.1	**0.01**
V1load	22.5 ± 5.1	**0.00**	14.8 ± 4.5	**0.01**
PF-RFD	10.9 ± 8.9	**0.02**	10.5 ± 8.7	**0.03**
T200m	−2.8 ± 1.1	**0.00**	−3.1 ± 1.2	**0.00**
T800m	−2.1 ± 0.8	**0.01**	−2.3 ± 0.9	**0.01**

The bold values denote p < 0.05.

**TABLE 3:** Pairwise Comparisons of Variables Between Groups at baseline (T1) and Post-training (T3).

Variables	Baseline			Post-training		
	HL-RT	BFR	*P*-values	HL-RT	BFR	*P*-values
1 RM (kg)	120.4 ± 11.9	118.6 ± 3.2	0.48	145.2 ± 12.4	134.6 ± 13.5	0.53
T20m (s)	3.15 ± 0.14	3.18 ± 0.16	0.38	3.04 ± 0.11	3.08 ± 0.14	0.41
CMJ (cm)	45.2 ± 4.1	44.8 ± 3.8	0.79	48.3 ± 4.2	47.7 ± 4.0	0.82
V1load (kg)	75.4 ± 8.2	74.1 ± 9.1	0.72	92.4 ± 8.5	85.1 ± 9.3	**0.04**
PF-RFD (N·s^-1^)	4850 ± 510	4790 ± 540	0.83	5380 ± 520	5290 ± 550	0.91
T200m (s)	24.4 ± 0.8	24.3 ± 0.7	0.70	23.2 ± 0.6	23.3 ± 0.5	0.49
T800m (s)	112.5 ± 3.1	113.1 ± 2.8	0.63	110.8 ± 2.5	111.2 ± 2.4	0.59

The bold values denote p < 0.05.

**FIGURE 1:**
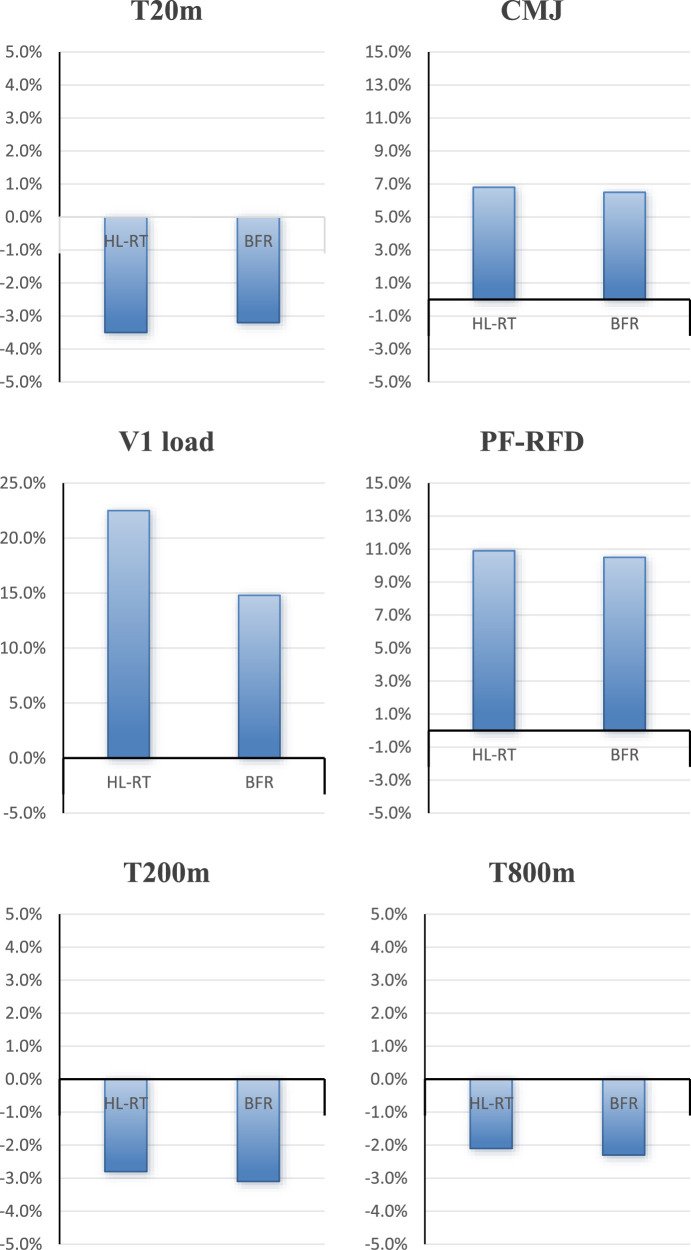
Percentage changes from pre-to post-intervention for key performance variables.

Intra-group comparisons revealed that, compared to pre-training, the HL-RT group exhibited significant improvements in several performance measures. Specifically, there were notable changes in 1RM (20.9% ± 8.4%), T20m (−3.5% ± 0.8%), CMJ (6.8% ± 2.9%), V1load (22.5% ± 5.1%), PF-RFD (10.9% ± 8.9%), T200m (−2.8% ± 1.1%), and T800m (−2.1% ± 0.8%), all of which were statistically significant (P < 0.05) (Figure 1; Table 2). Similarly, in the BFR group, significant improvements were also observed compared to pre-training in 1RM (14.3% ± 13.4%), T20m (−3.2% ± 1.7%), CMJ (6.5% ± 3.1%), V1load (14.8% ± 4.5%), PF-RFD (10.5% ± 8.7%), T200m (−3.1% ± 1.2%), and T800m (−2.3% ± 0.9%), with all changes reaching statistical significance (P < 0.05) (Table 2).

Baseline comparisons showed no significant difference in any variables in the groups before the training program (Table 3). Subsequent inter-group comparisons post-training revealed that, compared to the HL-RT group, the BRF group exhibited a significant increase in V1load, with statistical significance (P < 0.05). However, no statistically significant differences were observed in the other performance measures between the groups (Table 3).

## 4 Discussion

The results of this study demonstrated that (i) both HL-RT and BFR training significantly improved T20m, CMJ, PF-RFD, T200m, and T800m in college 800-m runners, with no significant difference between their effects; (ii) both HL-RT and BFR training significantly improved V1load, with HL-RT showing a significantly greater improvement training. These findings indicate that 8-week BFR training is as effective as traditional HL-RT in enhancing performance and running times in 800-m runners, with HL-RT proving more effective in improving muscular power.

Specifically, traditional HL-RT significantly improved the T20m, CMJ, V1load, and PF-RFD by 3.5%, 6.8%, 22.5%, and 10.9%, respectively, which is consistent with previous research (Ivey et al., 2000; Kosek et al., 2006). This finding indicates that even well-trained athletes can effectively enhance lower limb muscular strength and power through HL-RT, particularly when training loads exceed 60% of 1RM, as previous studies suggest significant improvements at this intensity (Peterson et al., 2004; Peterson et al., 2005). HL-RT recruits more high-threshold motor units, and activating these units helps maximize performance improvement (Kraemer and Ratamess, 2004). At the same time, our study found that BFR training also significantly improved the T20m, CMJ, V1load, PF-RFD, with increases of 3.2%, 6.5%, 14.8%, and 10.5%, respectively. BFR training creates an ischemic and hypoxic environment in the muscles, resulting in high levels of metabolic stress. This stress promotes both muscle protein synthesis and breakdown (Fry et al., 2010; Laurentino et al., 2012), enhances the recruitment of fast-twitch muscle fibers (Yasuda et al., 2009; Yasuda et al., 2013), and consequently leads to significant improvements in muscular strength and power.

In this study, BFR training resulted in performance improvements in the T20m, CMJ, and PF-RFD that were comparable to those observed with HL-RT. The results suggest that BFR training at 30% 1RM can produce similar effects to traditional RT at 80%–85% 1RM, which is similar to previous research results (Bielitzki et al., 2021; Loenneke et al., 2012). This supports the notion that BFR training can effectively enhance performance even when using reduced loads. However, as hypothesized, HL-RT showed significantly greater improvement in V1load, compared to BFR training (22.5% vs. 14.8%), highlighting the superior benefits of HL-RT in enhancing muscular power. Therefore, while BFR training offers significant training advantages with lower loads, HL-RT remains the preferred option for individuals primarily focused on increasing muscular power (Lixandrão et al., 2018; Geng et al., 2024).

Additionally, this study directly evaluated the running performance of athletes through T200m and T800m. Both BFR training and HL-RT significantly improved performance in these two events, further validating the effectiveness of both training methods. Meanwhile, no significant difference was observed between the two groups in either the T200m or T800m, indicating that the advantage of HL-RT in V1load did not translate into better running performance. This could be due to the fact that once maximal strength surpasses a certain threshold, it may negatively impact endurance performance, including decreased muscular endurance and metabolic efficiency (Marcora, 2009), thereby affecting overall performance. In comparison to HL-RT, BFR training offers a promising alternative for middle- and long-distance runners, as it can help maintain or even enhance muscular strength, power, and endurance without negatively affecting competition performance. BFR-induced metabolic stress, marked by metabolite accumulation and local hypoxia, closely mirrors the physiological demands of an 800-m race (Pearson and Hussain, 2015). This stress fosters peripheral adaptations, such as enhanced buffering and fatigue resistance, critical for race performance. Additionally, BFR promotes myogenic stem cell and myonuclei proliferation, supporting muscle adaptation and repair with reduced mechanical damage (Nielsen et al., 2012). Thus, BFR training stimulates muscle growth at lower loads, accelerates recovery, and helps prevent fatigue accumulation, ultimately enhancing training quality. This makes BFR training particularly suitable for athletes who are prone to injury or those who are unable to handle the higher loads (70%–80% 1RM) typically used in traditional strength training.

This study has several limitations. Firstly, the sample size was relatively small, consisting of only 22 participants, which may increase the influence of individual variations on the results. Secondly, this study was conducted exclusively with male runners, meaning the findings may not be applicable to female runners, as their response to RT could differ (Hawley et al., 2023). Thirdly, weather factors such as wind speed may influence the running test results. Although the tests were completed within a 3-h window, weather variations before and after the intervention were not considered. To enhance the robustness and generalizability of the findings, future research should aim to include a larger sample, further explore the application effects in female runners, and monitor relevant weather variables during the three testing sessions. Additionally,and the frequency of 1RM testing every second week may have been frequent enough to provide a strength stimulus to the participants, potentially exaggerating their outcomes. This represents an important limitation of this study.

## 5 Conclusion

Low-load BFR training offers an effective alternative to traditional HL-RT for enhancing competitive performance and key physical attributes in collegiate 800-m runners. HL-RT is ideal for those focusing on muscular power, while BFR training benefits runners prone to injury or unable to handle high loads in traditional strength training.

## Data Availability

The raw data supporting the conclusions of this article will be made available by the authors, without undue reservation.
